# α-Synuclein Expression Selectively Affects Tumorigenesis in Mice Modeling Parkinson's Disease

**DOI:** 10.1371/journal.pone.0019622

**Published:** 2011-05-18

**Authors:** Eitan Israeli, Eugenia Yakunin, Yonaton Zarbiv, Amir Hacohen-Solovich, Haya Kisos, Virginie Loeb, Michal Lichtenstein, Tziona Ben-Gedalya, Ofra Sabag, Eli Pikarsky, Haya Lorberboum-Galski, Ronit Sharon

**Affiliations:** 1 Department of Biochemistry and Molecular Biology, IMRIC, Hebrew University-Hadassah Medical School, Jerusalem, Israel; 2 Developmental Biology and Cancer Research, IMRIC, Hebrew University-Hadassah Medical School, Jerusalem, Israel; Brigham and Women's Hospital, Harvard Medical School, United States of America

## Abstract

Alpha Synuclein (α-Syn) is a protein implicated in mechanisms of neuronal degeneration in Parkinson's disease (PD). α-Syn is primarily a neuronal protein, however, its expression is found in various tumors including ovarian, colorectal and melanoma tumors. It has been hypothesized that neurodegeneration may share common mechanisms with oncogenesis. We tested whether α-Syn expression affects tumorigenesis of three types of tumors. Specifically, B16 melanoma, E0771 mammary gland adenocarcinoma and D122 Lewis lung carcinoma. For this aim, we utilized transgenic mice expression the human A53T α-Syn form. We found that the in vivo growth of B16 and E0771 but not D122 was enhanced in the A53T α-Syn mice. The effect on tumorigenesis was not detected in age-matched APP/PS1 mice, modeling Alzheimer's disease (AD), suggesting a specific effect for α-Syn- dependent neurodegeneration. Importantly, transgenic α-Syn expression was detected within the three tumor types. We further show uptake of exogenously added, purified α-Syn, by the cultured tumor cells. In accord, with the affected tumorigenesis in the young A53T α-Syn mice, over- expression of α-Syn in cultured B16 and E0771 cells enhanced proliferation, however, had no effect on the proliferation of D122 cells. Based on these results, we suggest that certain forms of α-Syn may selectively accelerate cellular mechanisms leading to cancer.

## Introduction

PD is a progressive, age-related neurodegenerative disorder, primarily defined by its related movement disabilities, including resting tremor, muscle tone rigidity and bradykinesia [1]. As the disease progresses, it also affects multiple systems within the central and peripheral nervous systems, and causes additional non-motor symptoms [2]. Pathologically, PD is characterized by dopaminergic neuronal loss in the nigro-striatal pathway of the brain and by the presence of Lewy bodies and Lewy neurites (reviewed in [3]) that with disease progression, spreads from the brain stem to the frontal neocortex [4]. Lewy bodies are intra-neuronal cytoplasmic inclusions whose primary structural component is α-Synuclein (α-Syn) (reviewed in [3]). α-Syn is a presynaptic protein critically involved in the cytopathology and genetics of PD (reviewed in [5], [6], [7]) and the related human synucleinopathies [8]. A progressive conversion of the soluble α-Syn protein, into soluble oligomers and insoluble aggregates is preceding its intraneuronal cytoplasmic deposition and underlies its cytopathology in this group of disorders [9], [10]. This progressive conversion and accumulation in cytotoxic forms of α-Syn is associated with neurodegeneration in PD [11].

The physiological role of α-Syn is still unclear. Recent studies have suggested that the neuronal α-Syn protein is involved in aspects of vesicle trafficking, both in exocytosis [12], [13], [14], [15], [16] and endocytosis [17]. In accordance with the findings indicating its enrichment in presynaptic terminals, α-Syn was shown to affect synaptic vesicle recycling [17], [18] and synaptic vesicle pools [19], [20].

Nevertheless, α-Syn expression is not restricted to the central nervous system. A portion of α-Syn is detected in plasma [21], red blood cells [22] and skin fibroblasts [23]. In addition, α-Syn expression was found in a variety of brain tumors [24], [25] as well as peripheral cancers, including ovarian and breast [26], colorectal tumors [27] and in melanoma [28].

It has been suggested that neuronal loss in neurodegenerative diseases may result from activation of cell cycle components. Neurons are generally considered to be post mitotic cells and are non-replicating. However, specific components of cell cycle machinery may be reactivated in some neurons upon certain stimuli (reviewed in [29]). While such activation of cell cycle machinery may result in proliferation of cancer cells, reactivation of cell cycle in post mitotic neurons may lead to apoptosis. Growing number of factors has recently emerged as being critically involved in both cancer and neurodegenerative diseases (reviewed in [30]).

In this study we aimed at testing whether α-Syn expression is involved in tumorigenesis. We tested the effect of α-Syn expression on the in vivo growth and proliferation of three types of murine tumors. Specifically, B16 melanoma, E0771 mammary gland adenocarcinoma and D122 Lewis lung carcinoma, utilizing the A53T α-Syn transgenic mouse model [31]. We found enhanced growth and proliferation for B16 and E0771 in this transgenic mouse model. Yet, no effect on tumorigenesis of D122 was detected. Importantly, we detected the transgenic, human, α-Syn expression within tumor cells and suggest, that these tumor cells uptake α-Syn which in turn, specifically activates certain cellular mechanisms leading to tumorigenesis.

## Materials and Methods

### Mice

The following mouse lines were used: C57BL6 (B6); APP/PS1 [32] (Jackson Laboratories, Bar Harbor, MN, USA); α-Syn null −/− mice C57BL/6JolaHsd [33]; C3H; F1 C57BL6/C3H (B6/C3HF1) (Harlan Laboratories, Rehovot, Israel); and A53T α-Syn +/+ [31]. α-Syn −/− and APP/PS1 harbor the B6 genetic background [34]; and A53T α-Syn+/+ harbor the B6/C3H genetic background.

This study was carried out in strict accordance with the recommendations in the Guide for the Care and Use of Laboratory Animals of the National Institutes of Health. The protocol was approved by the Committee on the Ethics of Animal Experiments of the Hebrew University of Jerusalem NIH approval # OPRR-A01-5011 (Permit Number: MD-09-11816-5). Surgery was performed under anesthesia with isofluran and all efforts were made to minimize suffering.

### Cells

The following B6-compatible cell lines were used: B16 melanoma [35], E0771 (obtained from Prof. Sirotnak F.M., Memorial Sloan-Kettering, New York, NY), and D122 [36]. B16 cells were grown and maintained in a Dulbecco's-modified Eagle's medium (DMEM) with 10% fetal calf serum, 2 mM L-glutamine and 100 U/ml Penicillin and Streptomycin (Biological Industries Ltd, Beit Haemek, Israel). For D122 cells, the conditioning medium included also 1 mM sodium pyruvate and MEM-non-essential amino acids solution (×1) (Biological Industries Ltd, Beit Haemek, Israel). E0771 cells were grown and maintained in RPMI 1640 supplemented with 10% fetal calf serum with iron (Biological Industries Ltd, Beit Haemek, Israel) and 10 mM HEPES. Stable poly-clones (stably expressing, non-clonal cultures) were generated with human wt α-Syn cDNA [37], human β-Syn cDNA [38] or amyloid precursor protein carrying the Swedish mutation (APPsw) [39] and the relevant mock-transfected clones, carrying the corresponding selection marker.

### α-Syn uptake by cultured cells

For uptake, cover slips were pretreated with poly-D-lysine (1%). Cells grown on cover slips for 24 hours, were incubated for 16 hours longer in standard conditioning medium, supplemented with purified α-Syn protein at a final concentration of 1 µM. Following the incubation with α-Syn protein, cells were washed twice and fixed. Immunocytochemistry was performed as previously described [37].

### Tumor growth and proliferation

To measure growth and proliferation we utilized a cohort of 3–4 or 9–10 months old A53T α-Syn+/+ and age-matched control mice (males and females, separated). Mice were irradiated at a dose of 500 rad one day prior to injections to suppress their immune response. On the day of injection, cells were washed twice in phosphate buffered saline (PBS), and counted. The mice were subcutaneously injected with the indicate number of cells (in 100 µl PBS) at the right-side of their lower back. Pilot experiments were performed in order to determine the number of cells for injection in each cell line. The factors tested involved measurable proliferation and ethical issues concerning tumor overgrowth. The mice were observed daily for tumor growth and general behavior. Tumor volume was measured independently by two researchers who were blinded to mouse genotype and was calculated as longitudinal diameter×lateral diameter^2^×0.4. Mice were sacrificed when the tumor reached 1.2 cm in any one dimension and final tumor weight was measured blinded to mouse genotypes.

### Cell proliferation assay

The CellTiter-Blue® (Promega, Beit Haemek, Israel), was used according to the manufacturer's instructions. Briefly, 5×10^3^ cells were seeded per well in a 96 well plates. Proliferation was measured at 24, 48 and 72 hours post seeding. At each time point, 20 µl of CellTiter-Blue® Reagent were added and incubated for one hour at 37°C. Then, the fluorescent signal was measured at 560 nm (excitation) and 590 nm (emission).

### Western blotting

Protein samples extracted from tumors in 1% NP-40 in PBS containing protease inhibitors cocktail (Sigma, Rehovot, Israel) were analyzed by a 10% SDS–PAGE. Immunoblots were reacted with the anti human α-Syn antibody, LB509 (Zymed); or with H3C antibody (generously provided by Prof. George J.M., University of Illinois) for both α-Syn and β-Syn detection; and 8E5 for APP detection. Immunoblots were normalized to the signal obtained for actin in the same sample (Sigma, Rehovot, Israel).

### Immunohistochemistry

Mice, anesthetized with an intraperitoneal overdose injection of Sodium Pentobarbitone (1 ml/1.5 kg), were perfused with phosphate-buffered saline (PBS). Following surgical removal of various tissues including the tumors, the tissues were kept frozen and used for further biochemical analyses or fixed for another 24 hr in the formalin. Histochemical analysis was performed with formalin fixed tissue as we have previously described [40]. Briefly, sections of (5 µm) were deparaffinized in xylene followed by graded alcohol in descending ethanol concentrations. Antigen retrieval was performed by incubating the slides in 100% formic acid for 5 minutes, followed by extensive washes. Sections were blocked in CAS-BLOCK (Invitrogen, Jerusalem, Israel). The sections were then immunostained using anti human α-Syn antibody LB 509 (1∶1000). Secondary ab was AlexaFluor-488 (1∶200, Molecular Probes). Staining were performed in parallel for the A53T α-Syn+/+ and control genotypes.

## Results

### Enhanced growth rates of B16 melanoma and E0771 adenocarcinoma but not D122 Lewis lung carcinoma in young A53T α-Syn +/+ mice

To investigate whether α-Syn expression affects tumor growth and proliferation we used a cohort of young, 3–4 month old, A53T α-Syn+/+ (a mix of four different litters) and control B6/C3H (F1 hybrid of C57BL6 and C3H genotypes) mice. Each mouse genotype was randomly divided in three groups (n = 5–8 mice in each group) and mice were subcutaneously injected either with B16 melanoma (2.5×10^5^), E0771 mammary gland adenocarcinoma (1×10^5^) or D122 Lewis lung carcinoma (5×10^4^) cells. An additional control group included PBS injected mice. Mice were assessed at least every alternate day for tumor size and the final tumor weight was determined at the day of termination, i.e., 15 days post injection. Tumor growth in the three cell-injected groups was progressive. No tumors were detected in the control PBS-injected group.

**Figure 1:**
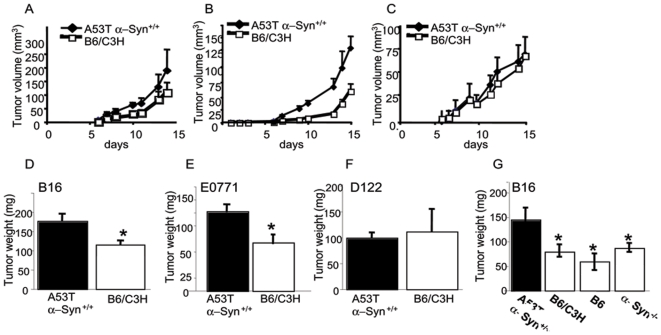
Tumorigenicity in young A53T α-Syn +/+ and control F1 B6/C3H mice. (a). Mice (3–4 months old) were subcutaneously injected with 0.25×10^6^ B16 melanoma cells. Tumor volumes were determined every 1–2 days post injection. The experiment was stopped after 15 days, when the tumors in the A53T α-Syn +/+ group reached 1 to 1.2 cm in diameter. The graph represents the means ± standard errors (SE) of 5–8 mice in each group. A representative growth curve out of three independent experiments. (b). Mice were injected with 1×10^5^ E0771 mammary gland adenocarcinoma cells and tumor volume was measured as in (a). (c). Mice were injected with 5×10^4^ D122 Lewis lung carcinoma and tumor volume was measured as in (a). (d). Mean weights of tumors formed in each genotype group at 15 days post- injection ± SE for B16, *,p = 0.015, t-test; (e). E0771, *, p = 0.012, t-test; (f). D122; (g). Mean weights of tumors formed in each genotype group at 15 days post- injection ± SE, n = 6–7, *, p<0.05 Mann-Whitney test.

The growth curves of B16 (Fig. 1a) and E0771 (Fig. 1b) tumors indicate significant higher growth rates in the A53T α-Syn+/+ than the control B6/C3H mice. Specifically, the mean ± SE growth in A53T α-Syn+/+ and control B6/C3H mice is: 3.8±0.7 and 1.2±0.3 mm^3^ per day (respectively) for B16; and 1.3±0.2 and 0.3±0.07 mm^3^ per day (respectively) for E0771. In contrast, no effect for transgenic α-Syn expression on D122 growth rate was detected (with mean ± SE growth set at 1.7±0.5 and 1.6±0.7 mm^3^ per day for A53T α-Syn+/+ and B6/C3H mice, respectively) (Fig. 1c). In agreement with the growth curves results, we measured a significant higher final tumor weight in A53T α-Syn+/+ than control B6/C3H mice for the B16 melanoma (178.4±24.4 and 113.5±10.2 mg, respectively, p = 0.015, t-test. Fig. 1d). Similarly, higher final tumor weight in A53T α-Syn+/+ than control B6/C3H is measured for E0771 (129.5±12.0 and 67.0±13.7 mg, respectively, p = 0.012, t-test. Fig. 1e); but not for D122 (98.3±11.1 and 112.4±19.8 mg, respectively, Fig. 1f) tumors.

Considering the accurate control genotype for the homozygous A53T α-Syn mice (originally generated with B6/C3H (F1)), we thought that the original B6/C3H (F1) mice may not fully represent an accurate genotype, as a genetic shift toward one of the parental genotype may have occurred during their constant breeding. We therefore injected B16 melanoma cells (as above) in C3H, B6 and B6/C3H control genotypes and compared tumor growth rates also in α-Syn −/− (B6 genetic background) and A53T α-Syn+/+. The C3H mice developed very small, poorly progressive B16 tumors (not shown). This result is expected because the B16 melanoma cells are known to be non-immunogenic and incompatible with the C3H mouse genotype [41]. Importantly, no differences in B16 tumor growth were detected between the two control genotypes, B6 and B6/C3H (F1) at any time point post injection (not shown) or in their final tumor weight (Fig. 1g). Specifically, the final tumor weight was 78.6±10.2 and 69.0±13.6 mg for B6/C3H and B6 genotype, respectively. Similarly, the final tumor weight for α-Syn−/− mice was not different than these two control genotypes, determined at 87.4±7.3 mg. In contrast, B16 melanoma tumors were significantly larger in the A53T α-Syn+/+, with a final mean weight of 167±22.7 mg (p<0.05 Mann-Whitney test).

### Tumor growth is not affected in old A53T α-Syn+/+ mice

**Figure 2:**
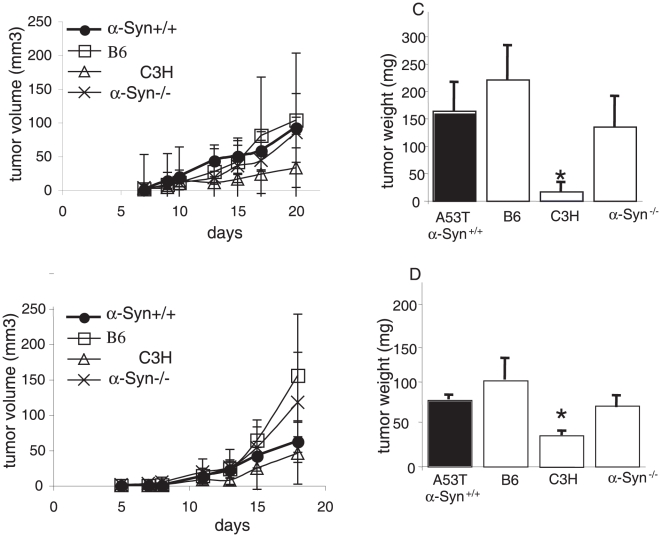
Tumorigenicity in old A53T α-Syn +/+ and control mice. (a). Mice (9–10 months old) were injected with B16 melanoma cells and tumor volumes were determined as in Fig. 1a. The experiment was stopped after 20 days, when the tumors in any genotype group reached 1 to 1.2 cm in diameter. The graph represents the means ± standard errors (SE) of 8–9 mice in each group. (b). Mice were injected with D122 Lewis lung carcinoma cells and tumor volume was measured as in Fig. 1a. Mean weights of tumors formed in each group at 20 days post- injection ± SE for B16 (c); and D122 (d). *, p<0.05 Mann-Whitney test.

To search for a potential association between α-Syn- related pathologies and tumor growth, we next measured the effect of α-Syn expression on tumor growth in old, symptomatic A53T α-Syn+/+ and control mice. Importantly, the A53T α-Syn+/+ mouse model involves an age-dependent neurodegeneration. Specifically, the mice appear generally healthy up to ∼7 month of age, at 9–10 month about 60% of the colony is symptomatic and at 12–15 month of age 100% of mice are sick (for a detailed description of this mouse model, see [31]). Cohorts of mice (including males and females, separated) at 9–10 month old A53T α-Syn+/+, and age-matched B6, C3H or α-Syn −/− control mice were randomly divided in two groups and injected in parallel, with B16 (2.5×10^5^) or D122 (5×10^4^ cells). Similar to the results obtained for young mice, poor proliferation of B16 was observed with the incompatible C3H control genotype (Fig. 2a–d). In addition, D122 tumor growth was not affected by the transgenic α-Syn expression in the old A53T α-Syn+/+ mice (Fig. 2b,d). However, unlike the results in young A53T α-Syn+/+ mice, the growth rate of B16 melanoma was not enhanced by the A53T α-Syn transgene. The growth curves and the final weight of B16 tumors were highly similar in the old A53T α-Syn+/+ and age-matched B6 control genotype (Fig. 2a,c). We therefore conclude that the effect of α-Syn expression on tumor growth is not associated with its level of pathogenesis.

### The proliferation of B16 melanoma is not affected in a mouse model for Alzheimer's disease

**Figure 3:**
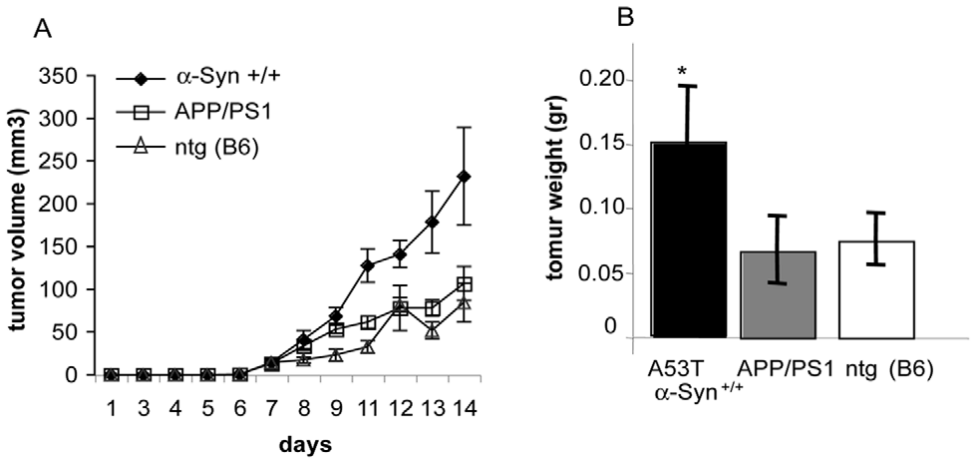
Tumorigenicity of B16 melanoma is not affected in APP/PS1 tg mice. (a). Mice (3–4 months old) were injected subcutaneously with 2.5×10^5^ B16 melanoma cells and tumor volumes were determined every 1–2 days post injection. The experiment was stopped after 14 days, when the tumors in the A53T α-Syn+/+ group reached 1 to 1.2 cm in diameter. The graph represents the means ± standard errors (SE) of 5–7 mice in each group. (b). Mean weights of tumors formed in each group at 14 days post- injection ± SE. *, p<0.05, Mann-Whitney test.

We next sought to determine whether the enhanced B16 melanoma growth rate in the young A53T α-Syn+/+ mice is specifically related to the transgenic α-Syn expression or rather, to general neurodegenerative mechanisms. For this aim we subcutaneously injected B16 melanoma cells (0.25×10^6^) in 3–4 month old APP/PS1+/− mice, modeling AD [34]; their non-transgenic littermates (harboring the B6 genotype); and age-matched A53T α-Syn+/+ mice. Tumor growth rates were measured (as above). Specifically, n = 5–7 mice in each group, containing males and females, separated. The results indicate very similar growth curves for the APP/PS1+/− and their non-transgenic littermates. Specifically, no differences in tumor initiation or its growth rate were detected between the two groups throughout the two weeks of measurements (Fig. 3a). Importantly, B16 melanoma growth rate in A53T α-Syn+/+ mice was significantly higher than the growth rates measured for the APP/PS1+/− and their non-transgenic control littermates, injected and treated in parallel (Fig. 3a). In accord, the final tumor weight was significantly higher in A53T α-Syn+/+ mice (149.4±43.2 mg) than APP/PS1+/− (65.1±26.2 mg) or control (B6 genotype) littermates (74.0±20.3 mg) (Fig. 3b).

### α-Syn expression is detected in the peripheral tumors

**Figure 4:**
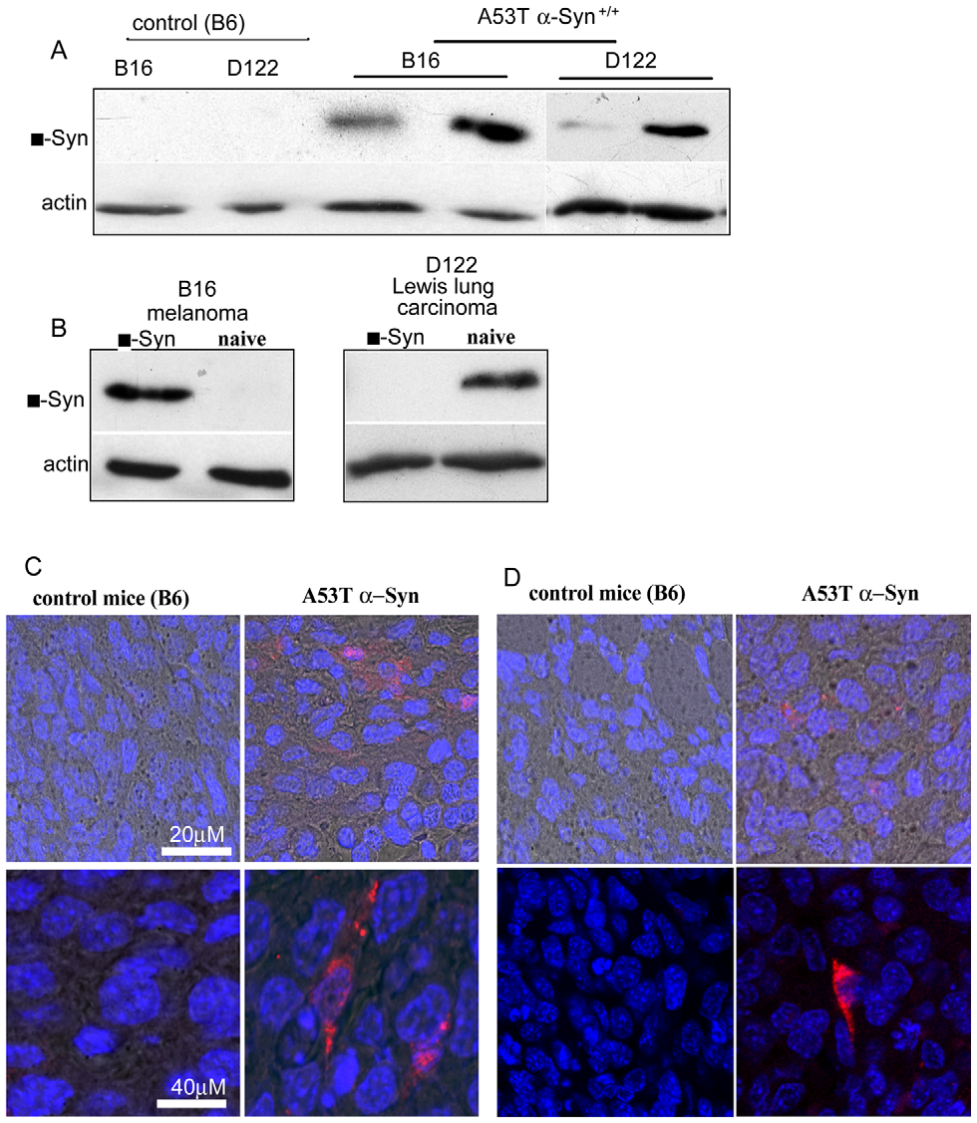
Human transgenic α-Syn is detected in B16 and D122 tumors from A53T α-Syn +/+ mice. (a). Protein samples (50 µg, detergent soluble) of B16 or D122 tumors from young control B6 and A53T α-Syn +/+ mice (each lane represents an individual mouse) analyzed by western blotting and probed with anti human α-Syn antibody, LB509. (b) Protein samples (50 µg, detergent soluble) of human α-Syn over expressing and naïve B16 melanoma or D122 Lewis lung carcinoma cells analyzed by western blotting as in (a). (c). IHC of Formalin-fixed B16 tumors from B6 and A53T α-Syn +/+ mice immunostained with the anti human α-Syn antibody, LB509 (red signal) (d) IHC of D122 tumors as in (c).

**Figure 5:**
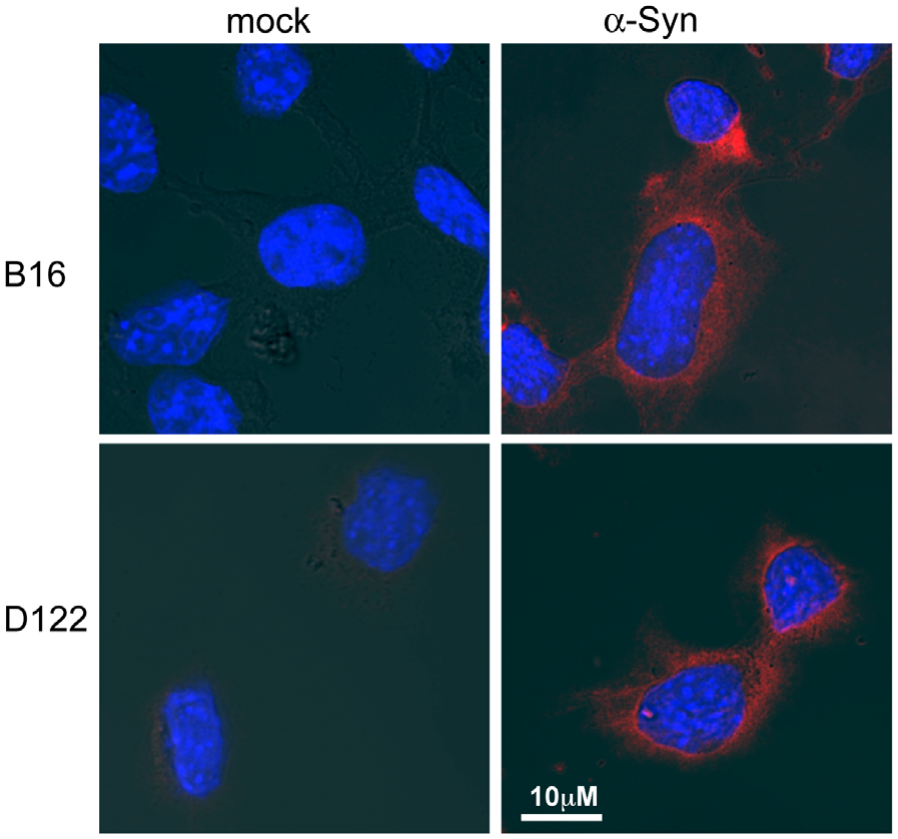
Culture B16 and D122 cells uptake exogenously added α-Syn. Cells were seeded on cover slips in 12 wells plates one day before the addition of purified α-Syn (1 µM) into standard serum-containing medium. Control sister cultures were grown and maintained in parallel but without the addition of recombinant α-Syn. Cells were then stained for the presence of α-Syn using anti human α-Syn antibody, LB509 (red signal).

Attempting to elucidate the mechanisms by which α-Syn, a neuronal protein affects the growth and proliferation of peripheral tumors, we searched for α-Syn expression within the tumors. For this aim, we analyzed protein samples extracted from the tumors of young (3–4 month old) A53T α-Syn+/+ and control B6 genotype by western blotting and probed with the anti human α-Syn antibody, LB509. We specifically focused on B16 melanoma and D122 Lewis lung canrcinoma, representing α-Syn -affected and -unaffected growth (respectively) (Figs. 4 and 5). Similar results were obtained for E0771 (not shown). Human, (transgenic) α-Syn immunoreactivity was detected in variable amounts in the different tumor types in the A53T α-Syn+/+ but not in control B6 mice (Fig. 4a). Importantly, no transgenic α-Syn signal was detected in the original injected cancer cells (Fig. 4b).

The occurrence of transgenic α-Syn in the tumors (which are of murine origin) may result from the PrP promoter, controlling the transgenic expression of A53T α-Syn, which is expressed in variable levels in non-neural tissues [42], including blood vessels in the tumors or connective tissues surrounding the tumors. In this case, the tumor sample may contain transgenic α-Syn that originated from the adjacent host transgenic mouse tissue. An alternative source for transgenic α-Syn in the tumors may be transgenic α-Syn protein, that has migrated from peripheral tissues or blood, into tumor cells. To specifically verify human transgenic α-Syn expression within tumor cells rather than connective tissues or blood vessels, we performed immunohistochemistry (IHC) on formalin sections of B16 and D122 tumors from A53T α-Syn+/+ and control B6 mice. We detected specific human transgenic α-Syn immunoreactivity in both, B16 and D122 tumors from A53T α-Syn+/+. No transgenic α-Syn immunoreactivity was detected in parallel-tested B16 or D122 tumors from control B6 mice (Fig. 4c,d).

### α-Syn uptake by cultured tumor cells

Growing evidence now suggest that a small portion of α-Syn is secreted from cells that express it and that this secreted α-Syn may than translocate into other, non neuronal cells. We sought to determine whether cultured tumor cells may uptake exogenously added α-Syn. For this aim, we incubated the different tumor cells, with a conditioning medium supplemented with purified human α-Syn (1 µM) and then performed immunocytochemistry using the anti human α-Syn, LB509 antibody. In parallel, we maintained sister cultures that were incubated in the same conditioning medium but without the addition of α-Syn protein. Following an incubation of 16 hours, a low but specific signal for human α-Syn was detected in B16 and D122 cells (Fig. 5). Similar result was obtained for E0771 cells (not shown). This result therefore suggests that the tumor cells uptake α-Syn protein from their surrounding environment.

### α-Syn expression enhances proliferation in cultured B16 and E0771 but not in D122 cells

**Figure 6:**
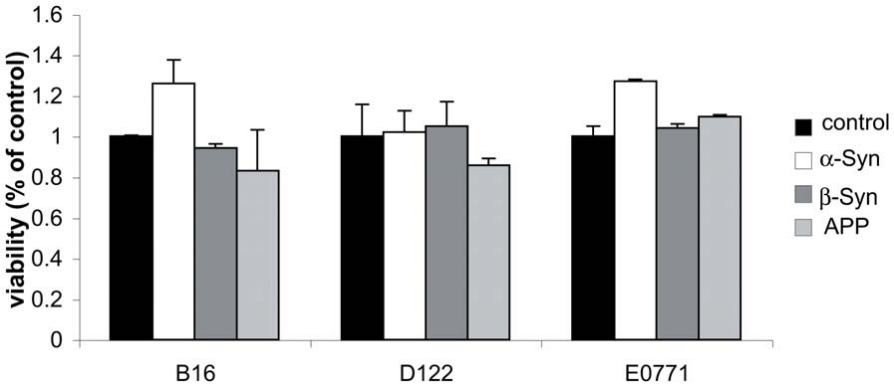
α-Syn expression in B16 and E0771 enhances cell proliferation. (a). Stable poly-clones of B16 cells over expressing either human wt α-Syn, human β-Syn, amyloid precursor protein carrying the Swedish mutation (APPsw) or mock-transfected, were seeded in a 96-well plates at 5×10^3^ cells per well. Proliferation was determined by the fluorescence ratio at 560ex/590em and normalized to the mock-transfected cells. A representative result of cells 48 hours post seeding. Mean ± SE of n = 6 wells out of three repeats. (b) Stable poly-clones of E0771 and (c) D122, seeded and measured as in (a). *, p<0.05, Mann-Whitney test.

We next sought to determine whether α-Syn expression affects cancer cell proliferation. For this aim, we generated stable poly clones of α-Syn over expressing B16, E0771 and D122 cells. For control cells, we generated poly clones over expressing β-Syn ; APP protein, carrying the Swedish mutation (APPsw); or mock transfected. Importantly, the expression level of the tested protein was highly similar between the different cell lines (not shown). The clones of each cell line were generated and maintained in parallel and α-Syn expression level was verified in the α-Syn expressing clones continuously [37]. The potential effect of over expressing these proteins on cell proliferation was determined after 24, 48 and 72 hours post seeding. The results indicate no effect for β-Syn or APP expression on cell proliferation after normalizing to the mock-transfected cells throughout the 72 hours of measurements. Specifically, no significant effect for β-Syn or APP expression was detected in neither one of the cell lines tested (i.e., B16, E0771 or D122 cells). However, a significantly higher rate of proliferation was observed upon α-Syn over expression in B16 and E0771 but not in D122 cells. Specifically, an increase of ∼25% in cell proliferation was detected post 48 hours (Fig. 6) and 72 hours (not shown) in B16 and E0771 cells over expressing α-Syn compared with the mock-transfected cells. These results suggest that α-Syn expression selectively affects cell proliferation in B16 and E0771, but not D122 cancer cells.

## Discussion

We tested whether α-Syn, a neuronal protein implicated in neuronal loss in PD and the related synucleinopathies, is involved in mechanisms of peripheral tumor growth and proliferation. We specifically tested the involvement of α-Syn expression in the growth and proliferation of murine B16 melanoma, E0771 mammary gland adenocarcinoma and D122 Lewis lung carcinoma. The specific cancer cell lines were injected subcutaneously and tumor growth rates and final tumor volumes were compared between the A53T α-Syn+/+ mice, modeling PD; APP/PS1, modeling AD; and control genotypes including, F1 B6/C3H, B6 and C3H. Our results indicate significantly higher growth rates for B16 and E0771 but not for D122 cancers in young, healthy, A53T α-Syn+/+ mice than in the different control genotypes tested. In accord with the effect on the growth rates, we measured a significant effect on final tumor volumes. That is, higher volumes were measured for B16 and E0771 but not D122 tumors grown in A53T α-Syn+/+ than control mice. Interestingly, detectable levels of human transgenic α-Syn protein were found within the tumor cells and we showed that cultured tumor cells uptake exogenously added α-Syn protein in their conditioning medium. In agreement with the in vivo results, over expressing α-Syn in cultured tumor cells selectively affected cell proliferation in B16 and E0771 but not in D122 cells. Together, these results suggest that α-Syn expression within the tumor cells specifically activates certain mechanism leading to tumorigenesis.

Emerging but still provocative data suggest that α-Syn is released from healthy neurons. Specifically, a small portion of α-Syn may be released via exocytic vesicles [43]. The secretion process may also occur with misfolded, cytotoxic forms of α-Syn, thereby allowing the “spread” of abnormal α-Syn to neighboring cells [44]. Despite these early findings, it remains unclear whether release of α-Syn is associated with its pathogenicity or whether it arises from a specific secretory pathway. No clear correlation has been reported between the levels of α-Syn in human plasma and CSF, and the occurrence of pathological α-Syn accumulation in PD patients [21], [44], [45], [46]. In this A53T α-Syn+/+ mouse model, the transgenic α-Syn expression is controlled by the prion protein promoter [31]. While the prion mRNA and protein are detected primarily in the CNS, it can also be found in peripheral tissue. Nevertheless, its expression is restricted to the host, transgenic mouse tissues. The finding of transgenic human α-Syn expression in the tumor cells, that are of non-transgenic mouse origin (Fig. 4), represent the potential translocation of human α-Syn from the host transgenic tissue to the non-transgenic tumor cells. Furthermore, the results indicating uptake of exogenously added α-Syn by cultured tumor cells (Fig. 5) further supports the explanation that human transgenic α-Syn released from neuronal cells in the CNS or peripheral cells may be the source of the human α-Syn detected within the tumors, acting to selectively affect their proliferation. Indeed, in our uptake experiments presented in Fig. 5, we have added a high amount of 1 µM of purified α-Syn to the conditioning medium. This amount is high considering that the estimated amount of α-Syn in plasma is ∼0.5–2 nM [21]. On the other hand, we exposed the tumor cells to exogenously added α-Syn for 16 hours, a short exposure time considering that the in vivo tumors are chronically exposed to the presence of α-Syn in the plasma. Importantly, α-Syn expression is routinely detected in various human tumors outside of the CNS. Including ovarian and breast [26], colorectal tumors [27] and in melanoma [28].

The results indicate a more aggressive growth for the tumor cells in the young (3–4 month) than the old (9–10 month) A53T α-Syn mice (Figs. 1 and 2). While α-Syn monomer is detected within the tumor cells in both, young (Fig. 4) and old mice (not shown), a thorough examination of the tumor cells is needed in order to better relate α-Syn toxicity to tumor proliferation. Specifically comparing for different α-Syn forms, including its post-translational modifications.

There is substantial evidence based on well-designed epidemiologic studies for generally low cancer rates in patients with Parkinson's disease (PD). Whereas the risk for leukemia, lymphoma colorectal, prostate and lung cancer appears to be lower, melanoma occurs more frequently among PD patients [47], [48], [49]. The mechanisms underlying the altered cancer morbidity among PD patients are not clear. Some speculations regarding the potential effect of levadopa or PD-related gene mutations have been suggested. However, the findings that the association between PD and cancer is present both before and after the diagnosis of PD and the rare occurrence of PD mutations, jeopardize these explanations [50].

PD is a multifactorial disease, involving at least 13 different genes and loci. Here we tested the potential involvement of α-Syn, one major factor implicated in the pathogenesis of PD, in mechanisms leading to tumorigenesis. The involvement of α-Syn in certain mechanisms leading to tumorigenesis, partly support the epidemiological findings indicating higher melanoma incidences among PD patients. Specifically, tumorigenesis of murine B16 melanoma was enhanced in A53T α-Syn+/+ mice compared with its growth and proliferation in the control genotypes. It is therefore called for cautiously relating this enhancing effect of α-Syn on proliferation of murine B16 melanoma to the increased incidences of melanoma among PD patients as documented by epidemiology. Nevertheless, our results do not provide an explanation to the reduced risk of various other cancer types, including, lung cancers as found by epidemiology.

We tested the effect of A53T α-Syn over expression in vivo and wt α-Syn over expression in cultured cells. Both wt and A53T mutant α-Syn over expression enhanced the proliferation of B16 melanoma and E0771 mammary gland adenocarcinoma. Similarly, both wt and A53T mutant α-Syn over expression had no effect on D122 Lewis lung carcinoma. The similar effects for wt and A53T mutant α-Syn described herein may suggest that the two proteins activate the same cellular mechanisms leading to enhanced tumorigenesis. Considering their critical role in PD [11], [51], it is possible that the two proteins act similarly in tumorigenesis and PD.

Dysregulation of genes that control cell cycle progression is a hallmark of tumorigenesis. In accord, an association between unscheduled cell cycle activity and neuronal apoptosis is related to neurodegenerative diseases such as AD [29], [52]. Growing evidence suggests that in post mitotic, fully differentiated neurons, attempts to induce proliferation results in apoptotic cell death and neurodegeneration (reviewed in [29]). In this regard, it is interesting to consider α-Syn as a protein involved in cell cycle control. We show that its over expression in B16 and E0771 cancer cells enhanced their proliferation. In contrast, α-Syn over expression in post mitotic neurons enhances mechanisms of cell death [53] and is known to cause neurodegeneration [11]. Therefore, although studies in neurodegenerative diseases and specifically, Parkinson's disease, might not usually be considered cancer research, they could ultimately provide insight into the function of genes and mechanisms associated with cancer, and help to characterize and refine biological pathways and/or therapeutic targets.

Additional PD-associated genes were shown to be involved in mechanisms leading to uncontrolled proliferation (reviewed in [54]). One example of a PD-gene that is involved in tumorigenesis is Parkin (PARK2). Parkin mutations are common among familial early onset PD and cause up to 50% of these cases [55]. Parkin, was long suspected to be a tumor suppressor gene because it resides on the long arm of chromosome 6, a segment that is known to be altered or deleted in a wide variety of human cancers and Parkin mutations were recently identified in a surprisingly large number of tumor types [56], [57], [58]. Parkin is an E3 ubiquitin ligase and its activity in marking proteins to degradation is implicated in the pathogenesis of PD. A recent study identified a novel Parkin E3 target protein, namely, cyclin E protein. In tumor cells containing Parkin mutations, cyclin E levels went up and cell proliferation was enhanced [56], [59]. Indeed, higher levels of cyclin E were reported in Parkin-deficient primary neurons and it was speculated that loss of Parkin causes dopaminergic neurons to re-enter the cell cycle and as a result, degenerate [60]. Based on the current knowledge, it is not expected that α-Syn and Parkin proteins share similar mechanisms in PD or cancer. However, the growing number of proteins affecting both neurodegeneration and cancer supports an association between unscheduled cell cycle activity and neuronal degeneration.

The synuclein family members include α, β and γ-synucleins, (α-Syn β-Syn and γ-Syn). While α-Syn and β-Syn have been specifically implicated in neurodegenerative diseases [51], [61], [62], [63], γ-Syn is not involved in neurodegeneration but primarily involved in tumoregenesis [26], [64], [65]. γ-Syn is highly expressed in breast carcinomas and usually predicts poor clinical outcome in breast cancer [66]. Interestingly, the initial reports describing increased incidences of cancer among PD patients specifically pointed at melanoma and breast cancer [47], [48]. However, a recent meta-analysis suggested that melanoma but not breast cancer occurs in high incidence among PD patients [49]. Investigations aimed to elucidate the molecular mechanisms underlying the oncogenic functions of γ-Syn revealed that γ-Syn expression in cancer cells results in an accelerated malignant phenotype with increased cell proliferation, motility, enhanced transcriptional activity and accelerated rate of chromosomal instability [64]. It is therefore interesting to find out whether α-Syn and γ-Syn, two proteins sharing ∼60% homology [10], affect similar cellular mechanisms leading to tumorigenesis.
